# Deciphering the Functional Composition of Fusogenic Liposomes

**DOI:** 10.3390/ijms19020346

**Published:** 2018-01-24

**Authors:** Rejhana Kolašinac, Christian Kleusch, Tobias Braun, Rudolf Merkel, Agnes Csiszár

**Affiliations:** Forschungszentrum Jülich GmbH, Institute of Complex Systems (ICS-7), Biomechanics, 52425 Jülich, Germany; r.kolasinac@fz-juelich.de (R.K.); Christian.Kleusch@nanotemper.de (C.K.); Tobi.braun@gmx.de (T.B.); r.merkel@fz-juelich.de (R.M.)

**Keywords:** membrane fusion, fusogenic liposomes, cationic lipid, aromatic compound, chromophore, neutral lipid

## Abstract

Cationic liposomes are frequently used as carrier particles for nucleic acid delivery. The most popular formulation is the equimolar mixture of two components, a cationic lipid and a neutral phosphoethanolamine. Its uptake pathway has been described as endocytosis. The presence of an aromatic molecule as a third component strongly influences the cellular uptake process and results in complete membrane fusion instead of endocytosis. Here, we systematically varied all three components of this lipid mixture and determined how efficiently the resulting particles fused with the plasma membrane of living mammalian cells. Our results show that an aromatic molecule and a cationic lipid component with conical molecular shape are essential for efficient fusion induction. While a neutral lipid is not mandatory, it can be used to control fusion efficiency and, in the most extreme case, to revert the uptake mechanism back to endocytosis.

## 1. Introduction

Positively charged liposomes have been well known in cell biology and biotechnology since the first cationic lipid *N*-[1-(2,3-dioleoyloxy)propyl]-*N*,*N*,*N*-trimethyammonium chloride (DOTMA) was synthesized by Felgner and co-workers and successfully used to introduce plasmid DNA to kidney cells [1]. This lipid-based transfection, also called lipofection, uses the attractive electrostatic interaction between the negatively charged nucleic acids and the positively charged liposomes to build liposome/DNA complexes [1,2]. Neutrally or negatively charged liposomes used before were not able to attach to nucleic acids and transfer them through cellular membranes into the nuclei of mammalian cells where the genetic information can be read out. Therefore, cationic liposomes became increasingly popular in biotechnology. Several research groups focused on the improvement of transfection efficiency by changing the liposomal composition, while others elucidated the lipid/DNA complex, called lipoplex, structures [2,3,4] and their cellular uptakes [5,6,7,8,9].

In the last decades novel cationic lipid analogues have been synthesized to minimize toxicity. At present, 1,2-dioleoyl-3-trimethylammonium-propane (DOTAP) is one of the most favoured and characterized cationic lipids [10]. In most cases, cationic lipids are used in combination with different neutral lipids or helper lipids, e.g., phosphoethanolamines, phosphocholines, or cholesterol. Such co-lipids at the same time decrease lipoplex toxicity and simultaneously increase transfection efficiency. It was observed by several research groups that the addition of a neutral lipid with a small head group and long, unsaturated fatty acid chains, e.g., 1,2-dioleoyl-3-glycero-phosphatidylethanolamine (DOPE), to the cationic lipids resulted in liposomes, that partially formed the inverted hexagonal (H_II_) phase in combination with the lamellar phases (L) [11]. In lipoplexes composed of the same lipid mixture and additional plasmid DNA H_II_ structures have been found even more. The inverted hexagonal lipid phase, together with other 3D phases, like hexagonal or cubic phases, may serve as intermediate structures for membrane fusion [9,12]. Therefore membrane fusion has been postulated as the main uptake route of such lipoplexes. However, investigations of the last decades clearly show that endocytosis plays the key role for cellular uptake of cationic liposomes while the contribution of membrane fusion is negligible [6,13]. Considering the slow dynamics of endocytosis and the fact that this process delivers the molecules of interest to the lysosomes where most of them are degraded, the low efficiency of lipofection can be explained.

It was shown by Csiszar et al. that the addition of an aromatic molecule as a third component to the usual cationic and neutral lipid mixture (DOPE/DOTAP) dramatically improves the membrane fusion efficiency of liposomes with the plasma membrane of mammalian cells [14]. Due to their extraordinarily high fusion efficiency, such liposomes have been called fusogenic liposomes (FLs). They have been successfully used for the delivery of different kinds of molecules, e.g., nucleic acids [15], purified proteins [16], polyphenol [17], anti-cancer therapeutics [18], fluorescent lipids [19] or nanoparticles [20]. The authors analysed liposomes containing DOPE as neutral lipid, DOTAP as cationic lipid component at an equimolar ratio and in addition fluorescently labelled lipids at ca. 5 mol % [14]. Due to the fluorescent emission of such molecules their cellular uptake was easily monitored by fluorescence microscopy.

Unfortunately, until now no systematic analysis has been carried out on the relative importance of the different components for fusion induction. In this work we aim at filling this gap. Therefore we systematically varied the liposomal composition and the concentration of each component, the cationic lipid, the neutral helper lipid and the aromatic compound. Liposomal characteristics like hydrodynamic diameter and zeta potential distributions were determined by dynamic and electrophoretic light scattering, respectively. Liposomal uptake by Chinese hamster ovary cells (CHOs) was visualized by fluorescence microscopy while the fusion efficiency of liposomes, with the same cells, was quantified by flow cytometry. The main goal of our study was to elucidate the interplay of liposomal composition, physicochemical characteristics, and fusion capacity.

## 2. Results

### 2.1. Changing the Lipid Composition of FLs: The Importance of Cationic Lipids

**Figure 1:**
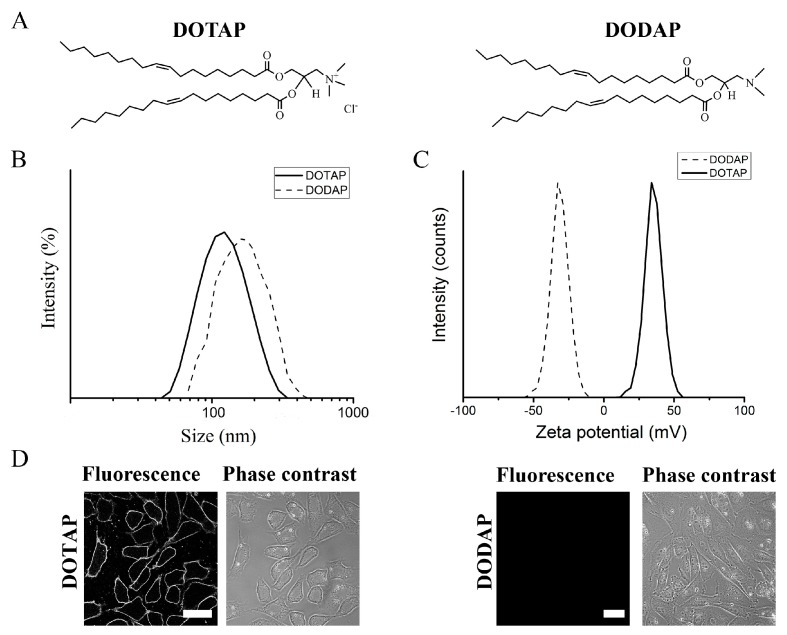
(**A**) Chemical structures of the positively charged lipid 1,2-dioleoyl-3-trimethylammonium-propane (DOTAP) and the neutral lipid 1,2-dioleoyl-3-dimethylammonium-propane (DODAP). (**B**) Hydrodynamic size and (**C**) zeta potential distributions of liposomes containing DOPE/DOTAP/BODIPY FL-DHPE and DOPE/DODAP/BODIPY FL-DHPE (1/1/0.1 mol/mol). (**D**) Fluorescence and phase contrast micrographs of CHO cells after treatment with liposomes containing DOTAP or DODAP. Scale bars, 20 µm.

The importance of positively charged lipids was investigated comparing two kinds of liposomes: one contained 1,2-dioleoyl-3-trimethylammonium-propane (DOTAP) a quaternary amine with +1 net charge in the head group range, the others contained 1,2-dioleoyl-3-dimethylammonium-propane (DODAP), a ternary amine without charge at pH 7.4 but with the same hydrophobic moiety (Figure 1A). These two lipids with comparable structures but different charges were embedded into liposomes additionally containing DOPE as neutral component and BODIPY FL-DHPE as aromatic component at a molecular ratio of DOPE/(DOTAP or DODAP)/BODIPY FL-DHPE = 1/1/0.1 mol/mol. Hydrodynamic size and zeta potential distributions of liposomes were measured using dynamic and electrophoretic light scattering (DLS), respectively. As shown in Figure 1B both liposomes had similar monomodal size distributions with maxima around 200 nm while their zeta potentials differed strongly. Liposomes containing DOTAP, a positively charged lipid, had a zeta potential of +41 mV (s.d. 10 mV) whereas liposomes with DODAP had a negative zeta potential of −30 mV (s.d. 10 mV) (Figure 1C). 

To test liposomal fusogenicity, Chinese hamster ovary (CHO) cells, as a typical mammalian cell line, were treated with the liposomes described above and liposomal uptake was visualized by fluorescence microscopy. Additional phase contrast micrographs yielded information about cell numbers and morphology (Figure 1D). When CHO cells were treated with liposomes containing DOTAP, a homogenous membrane staining was detected. This indicated complete mixing of liposomal and cellular membranes. In contrast to this, no liposomal uptake of CHOs was detected upon incubation with liposomes containing the neutral lipid DODAP. In both cases cell morphologies remained unchanged and appeared healthy.

**Figure 2:**
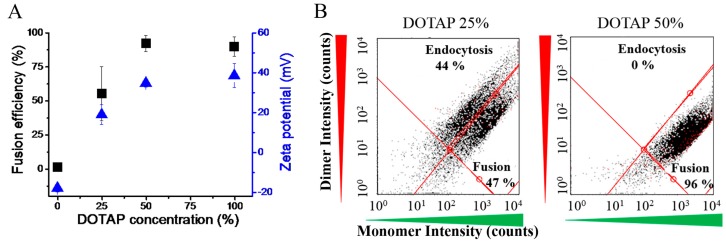
(**A**) Liposomal zeta potential (blue triangles) and fusion efficiency (black squares) at varying cationic lipid concentration. Bar indicate standard deviations. (**B**) Flow cytometry dot plots to determine the cellular uptake pathway and its efficiency. Liposomes always contained the aromatic tracer BODIPY FL-DHPE. Its monomer signal was detected in the green, its dimer signal in the red channels after incubation with Chinese hamster ovary cells (CHO). Endocytotic liposomal uptake resulted in a nearly equal dimer and monomer signals of the tracer, while a high monomer and a low dimer signal were detected in the case of membrane fusion.

In order to investigate the role of the positive charge of FLs in more detail, liposomes containing DOTAP as charged component, DOPE as helper lipid, and BODIPY FL-DHPE as aromatic component were prepared with varying DOTAP content between 0% and 95%. Zeta potentials were determined as previously described. Liposomes without DOTAP had a negative potential around −20 mV while increasing DOTAP concentration resulted in more and more positive values (Figure 2A). Similar zeta potentials were found at 50 mol % and 95 mol % of DOTAP content. This indicated saturation at about +40 mV.

We used the same liposomes for treatment of CHO cells and analysed membrane fusion efficiency by flow cytometry using the concentration dependent spectral changes of BODIPY FL. 

This technique allowed the statistical analysis of multiparametric data with high reliability. Moreover, it complemented the qualitative analysis of the liposomal uptake processes obtained by fluorescence microscopy. Instead of a Förster resonance energy transfer pair (FRET donor and acceptor) only one fluorescence dye, the BODIPY FL-DHPE, with concentration dependent spectral changes was used. When the labelled liposomes were internalized by membrane fusion, an intense emission peak appeared in the green spectral range (monomer signal) due to the dilution of the dye within the cellular plasma membrane. In the case of endocytic uptake, the BODIPY FL tracer remained at high local concentrations in the endosomes and was able to interact with each other building dimer pairs. This interaction influenced their spectral properties resulting in a red-shifted emission peak (dimer signal) that was absent at low concentrations (Figure 2B). We realized that fusion efficiency increased with increasing zeta potential of liposomes. Again, saturation occurred at a DOTAP concentration of about 50 mol %. Fusion efficiencies of approximately 90% were reached.

**Figure 3:**
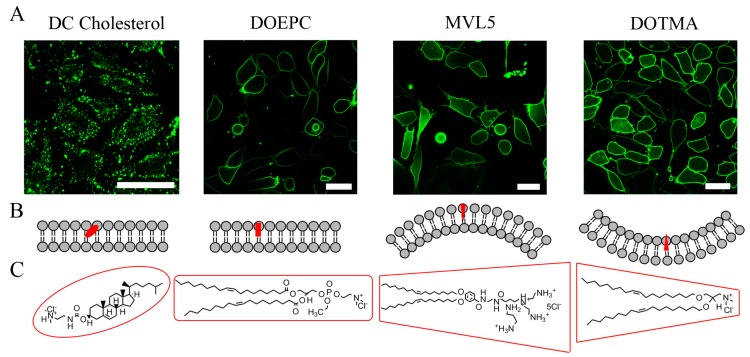
(**A**) Fluorescence micrographs of CHO cells after treatment with liposomes containing DC Cholesterol, DOEPC, MVL5 or DOTMA as cationic lipid, DOPE as neutral component and BODIPY FL-DHPE as fluorescent component (1/1/0.1 mol/mol). Scale bars, 20 µm. (**B**) Favoured membrane curvature of the reported cationic lipids. (**C**) Chemical structures of DC Cholesterol, DOEPC, MVL5 and DOTMA used as cationic lipids in FLs. Putative molecular shapes are indicated by red lines.

To explore the role of the positively charged component six different cationic lipids were embedded into FLs containing DOPE, cationic lipid and BODIPY FL-DHPE at a molar ratio of 1/1/0.1 mol/mol. These were DMTAP, DOTAP, DOTMA, DOEPC, MVL5, and DC-cholesterol (IUPAC names of lipids are listed in Section 2). Hydrodynamic diameters, zeta potentials, and fusion efficiencies of liposomes with CHO cells were determined as described above. The results are summarized in Table 1. While all liposomes had more or less the same sizes between 140 nm and 215 nm and zeta potentials between +30 mV and +40 mV, fusion efficiencies varied strongly. Liposomes containing the cationic lipids DOTAP or DOTMA were able to fuse with CHOs with efficiencies above 90%. In contrast to these, liposomes with DMTAP or DOEPC showed efficiencies of only ca. 30%. This value was even further reduced to 20% or even 0% in the case of liposomes with MVL5 and DC-cholesterol, respectively (Figure 3).

### 2.2. Changing the Lipid Composition of FLs: Importance of the Aromatic Component

**Figure 4:**
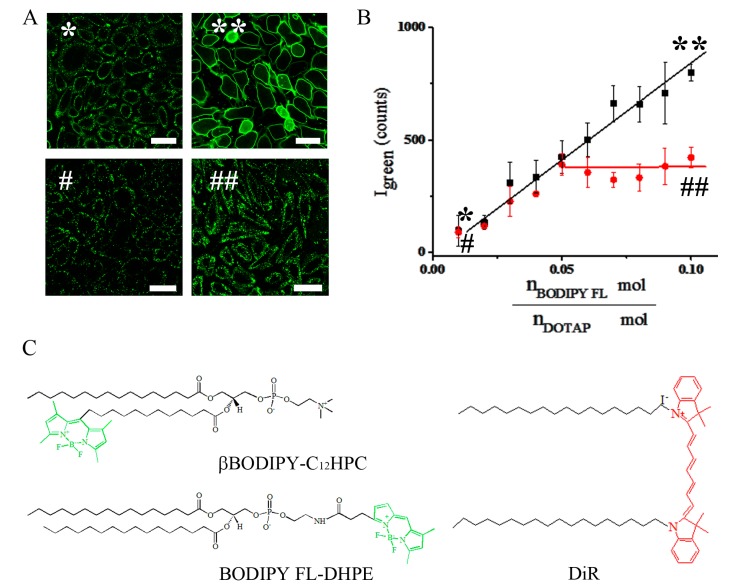
Importance of the aromatic component. (**A**) Fluorescence micrographs of CHO cells treated with liposomes containing BODIPY FL-DHPE as aromatic component in fusogenic liposomes (FL) at 0.01 (*) and 0.1 (**) mol/mol concentration as well as in endocytotic liposomes (EL) at the same concentrations (# and ##). Scale bars, 20 µm. (**B**) Dependence of fusion efficiency on dye concentration in FLs (black) and in ELs (red) determined by flow cytometry. The signal intensity median of the whole cell population was plotted vs. BODIPY FL-DHPE molar ratio to the cationic DOTAP amount in the liposomes (n_Bodipy FL-DHPE_/n_DOTAP_ mol/mol). Measurement points with standard deviations are shown as squares (FL) and circles (EL), respectively. Lines represent linear fits. (**C**) Molecular structures of the chain labelled lipid βBODIPY-C_12_HPC, the head labelled lipid BODIPY FL-DPHE, and the lipophilic membrane dye DiR incorporated in FLs as fluorescent components. The aromatic molecular parts are coloured green and red, representing their spectral emissions. Results for βBODIPY-C_12_HPC and DiR are shown in Supplementary Figures S1 and S2.

To test the role of the aromatic component, liposomes were prepared containing DOTAP as a cationic lipid, DOPE as a helper lipid (1/1 mol/mol) and BODIPY FL-DHPE, βBODIPY-C_12_HPC or DiR as aromatic component with varying amounts of the aromatic molecules. Total lipid ratios were set between 1/1/0.01 and 1/1/0.1 mol/mol. CHO cells were treated with these liposomes and fluorescence signal of dyes were observed by fluorescence microscopy. Depending on fluorophore concentration we observed two different characteristic staining patterns (Figure 4A and Figure S1A). A green speckled signal on the cell surfaces was recorded in the low BODIPY-FL-DHPE concentration range (0.01 mol/mol ≤ n_Bodipy FL-DHPE_/n_DOTAP_ ≤ 0.05 mol/mol) (Figure 4A: left image). This pattern was typical for endocytic uptake [21]. With increasing BODIPY FL-DHPE concentration, more and more cell shapes became visible due to homogenously stained plasma membranes upon membrane fusion and above a distinct dye concentration (n_Bodipy FL-DHPE_/n_DOTAP_ ≥ 0.05 mol/mol) all CHO cells showed completely stained plasma membranes (Figure 4A: upper right image). This signal was characteristic of highly efficient membrane fusion between liposomal and cellular membranes [21].

Based on the fluorescence signal of the BODIPY FL dye coupled to the DHPE lipid, the quantification of the internalized liposomal signal was carried out by flow cytometry. In the whole dye concentration range, signal intensity increased linearly with increasing BODIPY FL-DHPE concentration (slope 0.87 counts/dye ratio). Similar behaviour was detected when BODIPY FL-DHPE was replaced by a chain labelled lipid like βBODIPY-C_12_HPC or by carbocyanine fluorescent dye DiR (Figure 4C and Figures S1 and S2).

Liposomes with different composition were also analysed in the same way. Here, DOPE was replaced by DOPC and the concentration of the aromatic component was varied in the same range as described above. In the whole dye concentration range from 0.01 to 0.1 mol/mol, such liposomes resulted in the same speckled fluorescence pattern upon incubation with CHO cells (Figure 4A). Flow cytometry analysis revealed increasing signal intensity incorporated by cells with increasing dye concentration. The absolute intensity values obtained by DOPC containing liposomes were comparable with those containing DOPE in the low dye concentration range (0.01 mol/mol ≤ n_Bodipy FL-DHPE_/n_DOTAP_ ≤ 0.05 mol/mol), while they were significantly lower at higher dye concentrations (n_Bodipy FL-DHPE_/n_DOTAP_ ≥ 0.05 mol/mol) (slope 0.1 counts/dye ratio). The same trend was observed when BODIPY FL-DHPE was replaced by βBODIPY-C_12_HPC or by the carbocyanine fluorescent dye DiR (Figure 4C and Figures S1 and S2).

**Figure 5:**
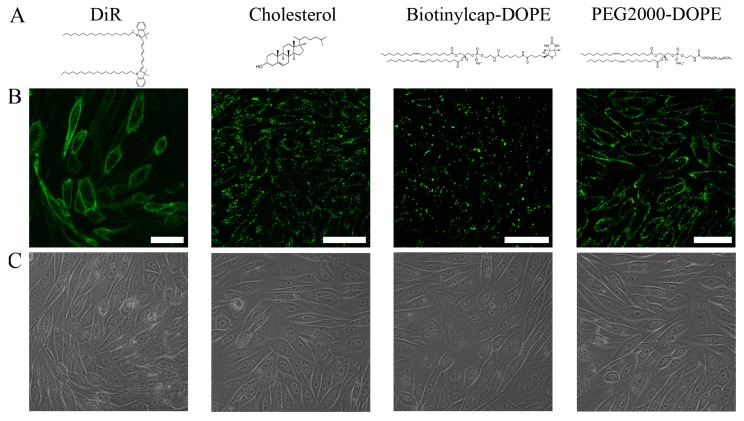
(**A**) Chemical structures of the aromatic DiR used as control, and the non-aromatic cholesterol, 1,2-dioleoyl-sn-glycero-3-phosphoethanolamine-*N*-(cap-biotinyl) (Biotinylcap-DOPE), and 1,2-dioleoyl-sn-glycero-3-phosphoethanolamine-*N*-[methoxy(polyethyleneglycol)-2000] (PEG2000-DOPE). (**B**) Fluorescence and (**C**) phase contrast images of CHO cells treated with liposomes containing the same components instead of an aromatic component, DOTAP as cationic lipid, DOPE as neutral lipid and BODIPY FL-DHPE as fluorescent tracer (0.1/1/1/0.02 mol/mol). BODIPY FL-DHPE was used at a non-fusogenic concentration. Scale bars, 50 µm.

To validate the importance of the aromatic component it was replaced by the non-aromatic molecules cholesterol, biotin and PEG2000 coupled to DOPE (Biotinylcap-DOPE and PEG2000-DOPE, respectively). For visualization BODIPY FL-DHPE was incorporated into the liposomes at a non-fusogenic concentration. Liposomes were composed as follows: DOPE/DOTAP/non-aromatic molecule/BODIPY FL-DOPE 1/1/0.1/0.02 mol/mol. In a control sample, DiR was used at its fusogenic concentration to release fusion and the fluorescence emission of BODIPY FL-DOPE was recorded in the green channel as shown in Figure 5. This pattern revealed a homogenously distributed green fluorescence in the plasma membrane of CHO cells, identified as a membrane fusion pattern (control sample). In all other cases green, speckled signals typical for endocytic processes were detected (Figure 5). Liposomal size and zeta potentials, however, were similar to those of fusogenic liposomes (see Table S2 in the Supplementary Materials). Nevertheless, these liposomes were not able to fuse with CHO cells.

### 2.3. Changing the Lipid Composition of FLs: The Importance of the Neutral Lipids

As shown above (Figure 2A), positively charged liposomes containing only a cationic lipid and an aromatic molecule, here a fluorescent dye, were able to fuse with the plasma membrane of mammalian cells. The additional helper lipid was not mandatory. However, neutral lipids can stabilize the formed liposomes and reduce cell toxicity. Therefore, we also investigated the influence of neutral lipids on cellular uptake pathway and fusion efficiency. For this purpose, we tested neutral lipids with different head groups, phosphocholine (PC), phosphoethanolamine (PE), and ceramide, with varying chain length from C14 to C22, as well as with saturated or unsaturated acyl chains (see Table 2).

Liposome composition was neutral lipid/DOTAP/BODIPY FL-DHPE 1/1/0.1 mol/mol. Liposomal size and zeta potential distributions were determined as described above. Moreover, liposomes were incubated with CHO cells and their cellular uptake and fusion efficiency were monitored by fluorescence microscopy and flow cytometry. All results are presented in Table 2.

#### 2.3.1. Effect of the Head Group

All investigated liposomes containing lipids with a phosphoethanolamine (PE) or phosphocholine (PC) as head group were analysed by DLS and any significant differences neither in size nor in zeta potential were found between PE and PC containing liposomes. 

**Figure 6:**
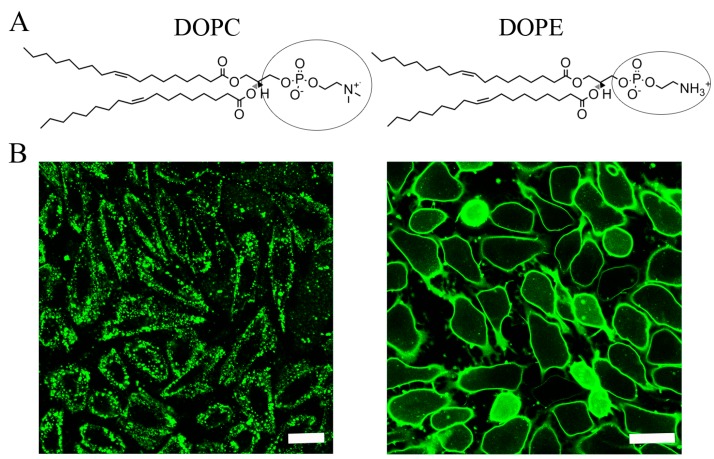
(**A**) Chemical structures of the neutral lipids 1,2-dioleoyl-*sn*-glycero-3-phosphocholine (DOPC) and 1,2-dioleoyl-*sn*-glycero-3-phosphoethanolamine (DOPE). (**B**) Fluorescence micrographs of CHO cells treated with liposomes containing DOPC (**left**) or DOPE (**right**) as neutral lipid, DOTAP as cationic lipid, and BODIPY FL-DHPE as fluorescent component (1/1/0.1 mol/mol). Scale bars, 20 µm.

**Figure 7:**
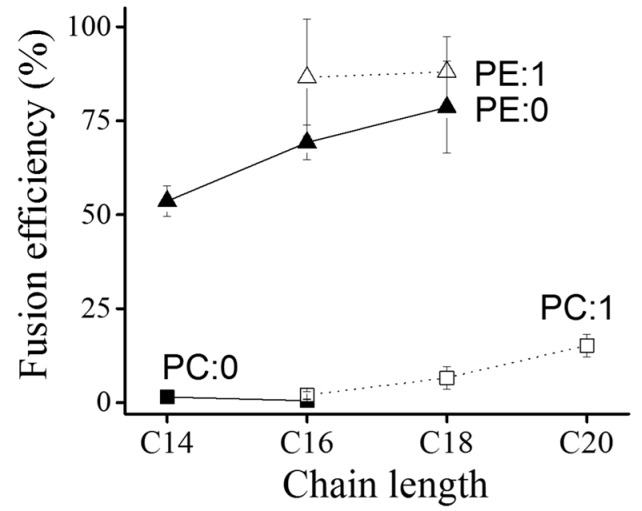
Liposomal fusion efficiency vs. neutral lipid component. Fusion efficiency of liposomes containing a neutral lipid component with different chain lengths and chain saturations as well as DOTAP and BODIPY FL-DHPE (1/1/0.1 mol/mol) was determined on CHO cells by flow cytometry. Symbols: saturated chains—filled symbols, unsaturated chains—open symbols, PCs—squares, PEs—triangles.

Subsequently, liposomal uptake mechanism and fusion efficiency were analysed. The chemical structure of DOPC as an example for PCs and DOPE for PEs are shown in Figure 6A. Liposomes containing these lipids were incubated with CHO cells and liposomal uptakes were visualized by microscopy. As shown in Figure 6B, in the case of DOPC a speckled signal of the fluorescent BODIPY FL-DHPE was detected. This clearly indicated endocytic cellular uptake. In contrast, a homogenous green fluorescent signal was observed in the plasma membrane of CHO cells when liposomes contained DOPE as neutral lipid. This fluorescent pattern proved membrane fusion between liposomes and cell membranes. Additional flow cytometry analyses revealed much higher fusion efficiency in the case of liposomes containing DOPE (87%, s.d. 8%) compared to those with DOPC (7%, s.d. 3%). Similar trends were noticed for all PCs and PEs tested (Table 2 and Figure 7) as well as for sphingolipids with small (ceramide) and large (sphingomyeline) head groups (Figure S3).

#### 2.3.2. Effect of Chain Length and Saturation

The influence of acyl chain length and saturation of the neutral lipid were also investigated. Neutral lipids with 14, 16, 18, and 20 carbon atoms (C14–C20), with or without one double bound in the acyl chains were used and the formed liposomes were characterized. Neither liposomal sizes nor zeta potentials varied systematically with chain length or saturation (Table 2). Additional flow cytometry analyses revealed that fusion efficiency increased with increasing acyl chain length. Moreover, chain unsaturation also increased the fusion ability of liposomes, especially in the case of neutral lipids with PE head groups (Figure 7). 

## 3. Discussion

Cationic liposomes in general are known as carrier particles for DNA plasmids and are frequently used for transfection. The most popular formulation has been the equimolar mixture of the cationic lipid DOTAP and the neutral phospholipid DOPE. Its uptake pathway has been described as mostly endocytosis [13,22]. Lipid molecules with a chromophore group added at ca. 5 mol % concentration to DOPE/DOTAP liposomes switched the liposomal uptake from endocytosis to membrane fusion [14]. In recent years, many applications of such liposomes have been established. In this endeavour it was noted that slightly varying liposomal compositions were needed to deliver different biological macromolecules to living cells [16,17,18,19,23,24]. However, a systematic study about the influence of liposomal composition on membrane fusion efficiency has still been missing. Therefore, we set out to systematically explore the role of the different molecules constituting of FLs. To do so, all three components, the cationic lipid, the neutral helper lipid and the chromophore, as well as their amount in the liposomes, were systematically varied, and the liposomes and their fusion ability were characterised.

### 3.1. Importance of the Cationic Lipid Component

Cationic lipids, in general, are not part of the natural lipid pool. They have been synthesized for the special application of transfection [1,25,26,27]. Some of them are listed in Table 1. Based on their attractive electrostatic interactions with negatively charged nucleic acids (DNA, mRNA, siRNA, etc.) they are able to complex such molecules. The most prominent cationic lipid 1,2-dioleoyl-trimethylammonium-propane (DOTAP) has been analysed here in combination with the neutral lipid DOPE and the fluorescently labeled BODIPY FL-DHPE with varying concentration between 0 and 95 mol %. DOPE liposomes, in general, are classified as fusogenic due to the conical effective molecular shape of DOPE. Although Siegel and co-workers showed fusion intermediate states by electron microscopy [12], the fusogenicity of DOPE alone seems to be insufficient for fusion with complex biomembranes. With increasing cationic lipid concentrations membrane fusogenicity increased (Figure 2). Notably, we found a correlation between liposomal fusion ability and positive zeta potential, as is characteristic for the liposomal surface charge. 

To elucidate the role of the cationic lipids in membrane fusion in more detail, different cationic lipids were analysed in the presence of a chromophore but without a neutral phospholipid like DOPE. We found marked differences in the fusion efficiencies of the respective liposomes although they all had similar zeta potentials (Table 1). These results can be understood from the molecular shapes of the cationic lipids. According to Kumar, membrane lipids can be classified into three general shape categories: inverted conical, cylindrical and conical [28]. The shape of a membrane lipid depends on the relative sizes of its polar head group and its apolar tails [29,30]. If head group and tails have similar cross-sectional areas, the molecule has a cylindrical shape. Lipids with a small head group and long unsaturated chains mostly have inverted conical shapes, while those chains occupy less area than their head groups when they are of conical shape [9]. We realised that the cationic lipids DOTAP or DOTMA with conical molecular shape were able to fuse with the cellular plasma membrane with efficiencies above 90%, while lipids with more cylindrical shapes like DMTAP or DOEPC fused with an efficiency of only ca. 30% with the cell membrane. This value was reduced even further to 20% if the cationic lipid had a rather inverted conical shape like MVL5. 

As shown in Figure 4 and described by Chernomordik and Kozlov [8,9,31], the tendency of lipids to form curved layers correlates with their effective molecular shapes. Molecules with a conical effective shape tend to form monolayers with negative curvature, which is a necessary prerequisite for the formation of the fusion intermediate state. 

### 3.2. Role of the Neutral Lipid Component

Most liposomal formulations used for gene delivery contain a neutral or helper lipid component besides the cationic lipids [32,33,34]. We could show that the presence of a neutral or zwitterionic lipid is not mandatory for membrane fusion (see Figure 2A). However, the presence of such lipids can strongly influence the liposomal uptake mechanism, as already shown by Braun et al. [21]. Here, DOPE was replaced as the neutral lipid by DMPC and endocytosis was detected as the main uptake pathway instead of membrane fusion. Intrigued by this strange effect, we systematically investigated the influence of the neutral component by varying lipid head group and acyl chains. Phosphocholine (PC), phosphoethanolamine (PE) and ceramide (CER) were tested. We observed increasing fusion abilities with decreasing head group size (Figure 7 and Figure S3). Liposomes containing PCs were almost unable to fuse, which generalizes the findings of Braun et al. [21]. Additionally, liposomes containing ceramide, the neutral lipid with the smallest head group tested in this study, fused so effectively that liposomal concentration had to be reduced to minimize toxicity (Figure S3). These results underline previous studies describing PEs, in general, and DOPE especially, as fusogenic lipids [19,27].

### 3.3. Importance of the Chromophore

Three different fluorophores were compared to elucidate the role of the chromophore. These were BODIPY FL-DHPE, a head labelled lipid, βBODIPY-C_12_HPC, a chain labelled lipid, and DiR, a lipid analogue, whom chromophore most likely resides in the lipid backbone range [35]. All three lipids were able to induce membrane fusion above a distinct concentration of 2.5 mol % (1/1/0.05 mol/mol). We suggest that the chromophores induce high local instabilities in the lipid membrane. These instabilities in a non-equilibrium membrane probably initiate the formation of fusion intermediate phases, e.g., inverted-hexagonal or cubic phases [9,12,36]. Still, neither the simple presence of phospholipids with conical effective molecular shape but without net positive charge, e.g., DOPE, in combination with the aromatic chromophore, nor the presence of a cyclic component without π-electron system is sufficient for fusion induction (Figure 2 and Figure 5). We hypothesize that electrostatic interaction between the positively charged lipids and the highly polarizable π-electron system of the fluorophores serves as additional interaction to finally induce fusion. Our hypothesis is supported by the facts that molecules with aromatic character, but weak emissions in the ultraviolet or visible spectral range like resveratrol or other polyphenols [17,37] are also able to induce membrane fusion while missing delocalized electrons as in, e.g., biotin, results in barely fusogenic liposomes.

## 4. Materials and Methods

### 4.1. Chemicals

As cationic lipid components the following molecules were used: 1,2-dimyristoyl-3-trimethylammonium-propane (chloride salt) (DMTAP), 1,2-dioleoyl-3-trimethylammonium-propane (chloride salt) (DOTAP), 1,2-dioleoyl-3-dimethylammonium-propane (DODAP), 1,2-di-*O*-octadecenyl-3-trimethylammonium propane (chloride salt) (DOTMA), 1,2-dioleoyl-*sn*-glycero-3-ethylphosphocholine (chloride salt) (DOEPC), 3β-[*N*-(*N*′,*N*′-dimethylaminoethane)-carbamyl]cholesterol hydrochloride (DC-cholesterol), and *N*1-[2-((1S)-1-[(3-aminopropyl)amino]-4-[di(3-amino-propyl)amino]butylcarboxamido)ethyl]-3,4-di[oleyloxy]-benzamide (MVL5). As neutral or helper lipids the following components were used: 1,2-ditetradecanoyl-*sn*-glycero-3-phosphoethanolamine (DMPE), 1,2-dihexadecanoyl-*sn*-glycero-3-phosphoethanolamine (DPPE), 1,2-distearoyl-*sn*-glycero-3-phosphoethanolamine (DSPE), 1,2-dipalmitoleoyl-*sn*-glycero-3-phosphoethanolamine (DPaPE), 1,2-di-(9Z-octadecenoyl)-*sn*-glycero-3-phosphoethanolamine (DOPE), 1-stearoyl-2-hydroxy-*sn*-glycero-3-phosphoethanolamine (Lyso PE), 1,2-dimyristoyl-*sn*-glycero-3-phosphocholine (DMPC), 1,2-dipalmitoyl-*sn*-glycero-3-phosphocholine (DPPC), 1,2-distearoyl-*sn*-glycero-3-phosphocholine (DSPC), 1,2-dipalmitelaidoyl-*sn*-glycero-3-phosphocholine (DPaPC), 1,2-dioleoyl-*sn*-glycero-3-phosphocholine (DOPC), 1,2-dieicosenoyl-*sn*-glycero-3-phosphocholine (DEPC), 1,2-dilinoeloyl-*sn*-glycero-3-phosphocholine (DLiPC), 1-oleoyl-2-hydroxy-*sn*-glycero-3-phosphocholine (Lyso PC), *N*-oleoyl-d-*erythro*-sphingosine ceramide (d18:1/18:1(9Z)) (CER), and *N*-oleoyl-d-*erythro*-sphingosylphosphorylcholine. As non-aromatic third components cholesterol (cholesterol), 1,2-dioleoyl-*sn*-glycero-3-phosphoethanolamine-*N*-(cap biotinyl) (sodium salt) (Biotinylcap-DOPE), and 1,2-dioleoyl-*sn*-glycero-3-phosphoethanolamine-*N*-[methoxy(polyethylene glycol)-2000] (ammonium salt) (PEG2000-DOPE) were used. All lipids described thuf ar were purchased from Avanti Polar Lipids, Inc. (Alabaster, AL, USA) and used without further purification. The fluorescently labelled lipids *N*-(4,4-difluoro-5,7-dimethyl-4-bora-3*a*,4*a*-diaza-*s*-indacene-3-propionyl)-1,2-dihexadecanoyl-*sn*-glycero-3-phosphoethanolamine (triethylammonium salt) (BODIPY FL-DHPE) and 2-(4,4-difluoro-5-methyl-4-bora-3*a*,4*a*-diazas-indacene-3-dodecanoyl)-1-hexadecanoyl-*sn*-glycero-3-phosphocholine (βBODIPY-C_12_HPC), and the lipid analogue DiI(C18)7 (DiR) were ordered from Thermo Scientifics (Eugene, OR, USA). 

### 4.2. Preparation of Liposomes

Liposomes were prepared according to the method described by Csiszár et al. [14] with few modifications. In brief, lipid components like neutral lipid, positively charged lipid and the fluorescent compounds were mixed in chloroform (VWR, Darmstadt, Germany) at a ratio of 1/1/0.01–0.1 mol/mol. All liposomal compositions tested here are summarized in Table S1 (Supplemental). Chloroform was evaporated under a vacuum for 0.5 h. Then, lipids were dispersed in 20 mM 2-(4-(2-hydroxyethyl)-1-piperazinyl)-ethansulfonic acid (HEPES) buffer (VWR, Darmstadt, Germany) at a total lipid concentration of 2 mg/mL at pH 7.4 (π = 70 mOsm). The solution was vortexed for 1–2 min to produce multilamellar liposomes. After homogenization in an ultrasonic bath (Sonocool, Bandelin electronic GmbH, Berlin, Germany) for 20 min at 5 °C, mainly unilamellar vesicles were formed. 

### 4.3. Characterization of Size and Zeta Potential Distributions of Liposomes Using Dynamic Light and Electrophoretic Light Scattering

Both, particle size and ξ-potential distributions were measured using a zetasizer (Nano ZS from Malvern Instruments, Malvern, UK) equipped with a HeNe laser (633 nm). Scattered laser light was collected at a constant angle of 173°. Prior to measurements liposome stock solutions were diluted 1/50 with purified, degassed and filtrated water (Milli-Q Gradient A10, Merck Millipore, Darmstadt, Germany). All measurements were performed at 20 °C and repeated three times at 1 min intervals. Data were collected from three independently prepared samples and analysed using the instrument software (DTS from Malvern Instruments). Reported data are mean peak position and its standard deviation (mean (s.d.)).

### 4.4. Cell Culture

Experiments were performed on Chinese Hamster Ovary K1 (CHOs) cells purchased from American Type Culture Collection (ATTC, Manassas, VA, USA). They were maintained in DMEM-F12 (Sigma-Aldrich, Taufkirchen, Germany) supplemented with 10% fetal bovine serum (FBS) and 10,000 units penicillin and 10 mg/mL streptomycin (Sigma-Aldrich). During culture as well as experiment, cells were kept at 37 °C and 5% CO_2_ in a saturated humid atmosphere. Cell density never exceeded 80% confluence. For microscopy and flow cytometry analyses 30,000 cells were seeded on fibronectin (BD Biosciences, San Jose, CA, USA) coated (10 µg/mL, 30 min) glass surfaces one day before the experiment.

Prior to experiments, 10 µL of the liposome stock solutions were diluted 1/50 with phosphate buffered saline (PBS) (Sigma-Aldrich). Cells cultivated on a Petri dish (Ø = 3.5 cm) were incubated in liposome solution (pH 7.4) for 5 min at 37 °C. Subsequently, liposome solutions were replaced by fresh medium and the internalized cellular fluorescence was analysed by light microscopy and flow cytometry.

### 4.5. Microscopy

Samples were imaged using a confocal laser scanning microscope (LSM 710 from Carl Zeiss MicroImaging GmbH, Jena, Germany) equipped with an argon ion laser (488 nm) and a HeNe laser (633 nm). Both BODIPY derivatives were exited at 488 nm and their fluorescence emissions were detected using a band pass filter BP 495–550 nm (green channel). The lipid analogue DiR was excited using the 633 nm laser line and the emitted signal was detected through the long pass filter LP 650 nm. For imaging a Plan-Apochromat 40×/1.40 Ph3 (Carl Zeiss) objective was used. To maintain appropriate culture conditions, the microscope was equipped with an incubator (Incubator XL 2, Carl Zeiss). Temperature and CO_2_ were kept constant at 37 °C and 5%, respectively. The images were analysed using ZEN software (Carl Zeiss).

### 4.6. Flow Cytometry

Internalized cellular fluorescence intensities were analysed using a flow cytometer (Guava easyCyte 8HT, Merck Millipore, Billerica, MA, USA) equipped with a guava Flowcell II (Merck Millipore) and a 75 mW argon-ion laser to excite the BODIPY fluorophores at 488 nm. The emitted monomer signal was measured using a 525/30-nm band-pass optical filter (green channel) while the dimer signal of this dye was collected using a 690/50-nm band-pass optical filter (red channel) [21]. For each sample a minimum of 10,000 cells were collected. Data were analysed using the InCyte Software for Guava easyCyte HT Systems (Merck Millipore). Prior to analysis, cells were trypsinized with a trypsin–EDTA solution (Sigma-Aldrich) for 5 min, subsequently washed with phosphate buffered saline (PBS) (pH 7.4) and centrifuged for 5 min at 200× *g*. Analyses were carried out either on living cells in PBS or on fixed cells. Cell fixation was carried out using a cell fixation reagent (Solution A from Fix&Perm cell permeabilization kit from Life Technologies, Carlsbad, CA, USA). All measurements were performed at least in three independent experiments in duplicates. Analysis was performed as described by Braun and co-workers [21]. Briefly, cells were selected and quantified using a two-dimensional dot plot of the forward scatter signal (FSC) vs. side scatter signal (SSC) (both logarithmic plots). Photomultipliers of the green (monomer) and red (dimer) channels were adjusted using untreated CHOs that the detected signal intensities remained below 10 counts. During analysis no compensation was required. “Endocytosis” and “Fusion” gates were determined based on the two-dimensional dot plot of green vs. red channels (both log plots) using endocytic and fusogenic liposomes, respectively, at a BODIPY concentration of 5 mol %. Each gate contained more than 90% of all events. 

### 4.7. Statistical Analysis

Statistical analyses of data were performed by one-way ANOVA using Origin 9.0 (OriginLab Co., Northampton, MA, USA). *p* < 0.01 was considered statistically significant. Data are expressed as means (s.d.).

## 5. Conclusions

Our study revealed that an aromatic molecule and a cationic lipid component with conical molecular shape are essential for efficient fusion induction. While a neutral lipid is not mandatory, it can be used to control fusion efficiency. The special role of the aromatic compound deserves further investigation, with special analysis of electrostatic interactions and molecular polarizability.

## Figures and Tables

**Table 1:** Characteristics of FLs containing different cationic lipids: molecular shape of lipids, fusion efficiency of liposomes with CHO cells, zeta potentials (ς) and hydrodynamic sizes (d) of liposomes containing the respective cationic lipid, DOPE as helper lipid and BODIPY FL-DHPE as fluorescent molecule (1/1/0.1 mol/mol). Efficiencies of endocytosis and fusion give together 100%. Average values of at least three independent measurements and their standard deviations are given.

Cationic Lipids	Molecular Shape	Fusion eff. (s.d.) (%)	ς (s.d.) (mV)	d (s.d.) (nm)
DOTAP	conical	92 (6)	41 (10)	214 (45)
DOTMA	conical	95 (4)	42 (12)	126 (36)
DMTAP	cylindrical	35 (7)	31 (6)	149 (39)
DOEPC	cylindrical	33 (6)	41 (19)	164 (8)
DC-Cholesterol	cylindrical	1 (1)	42 (1)	136 (1)
MVL5	inv. conical	20 (2)	26 (8)	152 (7)

**Table 2:** Characteristics of liposomes containing different neutral lipids, DOTAP as cationic component, and BODIPY FL-DHPE as fluorescent component (1/1/0.1 mol/mol): head group, chain length, number of double bonds, and molecular shape of the neutral lipid component, fusion efficiency of liposomes with CHO cells, as well as liposomal zeta potential (ς), and hydrodynamic size (d). Efficiencies of endocytosis and fusion give together 100%. Average values of at least three independent measurements and their standard deviations are given.

Lipid	Head Group	Chain Length	Double Bonds	Molecular Shape	Fusion eff. (s.d.) (%)	ς (s.d.) (mV)	d (s.d) (nm)
DMPE	PE	14	0	conical	54 (15)	62 (4)	242 (14)
DPPE	PE	16	0	conical	69 (5)	60 (5)	140 (20)
DPaPE	PE	16	1	conical	93 (5)	69 (2)	109 (24)
DSPE	PE	18	0	conical	79 (9)	67 (8)	170 (14)
DOPE	PE	18	1	conical	87 (8)	68 (1)	124 (31)
LysoPE	PE	18	1	inv.conical	4 (2)	65 (3)	99 (4)
DMPC	PC	14	0	cylindrical	1 (1)	55 (12)	141 (64)
DPPC	PC	16	0	cylindrical	1 (0)	57 (5)	140 (55)
DPaPC	PC	16	1	cylindrical	2 (1)	65 (8)	205 (60)
DSPC	PC	18	0	cylindrical	48 (11)	50 (4)	141 (4)
DOPC	PC	18	1	cylindrical	7 (3)	58 (9)	176 (96)
DLiPC	PC	18	3	cylindrical	56 (17)	55 (3)	121 (15)
DEPC	PC	20	1	cylindrical	15 (3)	56 (7)	183 (103)
LysoPC	PC	18	1	inv.conical	3 (1)	45 (1)	131 (46)
SM	PC	18	1	cylindrical	3 (1)	58 (4)	120 (25)
CER	OH	18	1	conical	88 (9)	56 (9)	154 (10)

## Supplementary Materials

The following are available online at www.mdpi.com/1422-0067/19/2/346/s1.
