# Supplementary material for: Deciphering the Functional Composition of Fusogenic Liposomes
Source: Int J Mol Sci. 2018 Jan 24;19(2):346. doi: 10.3390/ijms19020346 (PMC5855568; doi:10.3390/ijms19020346)
Supplement: Supplementary file 1 [file ijms-19-00346-s001.pdf]

International Journal of
Molecular Sciences

MDPI

Article

Deciphering the Functional Composition of Fusogenic Liposomes

Rejhana Kolašinac, Christian Kleusch, Tobias Braun, Rudolf Merkel and Agnes Csiszár *

Forschungszentrum Jülich GmbH, Institute of Complex Systems (ICS-7), Biomechanics, 52425 Jülich, Germany; r.kolasinac@fz-juelich.de (R.K.); Christian.Kleusch@nanotemper.de (C.K.); Tobi.braun@gmx.de (T.B.); r.merkel@fz-juelich.de (R.M.)

* Correspondence: a.csiszar@fz-juelich.de; Tel.: +49-2461-6114-15

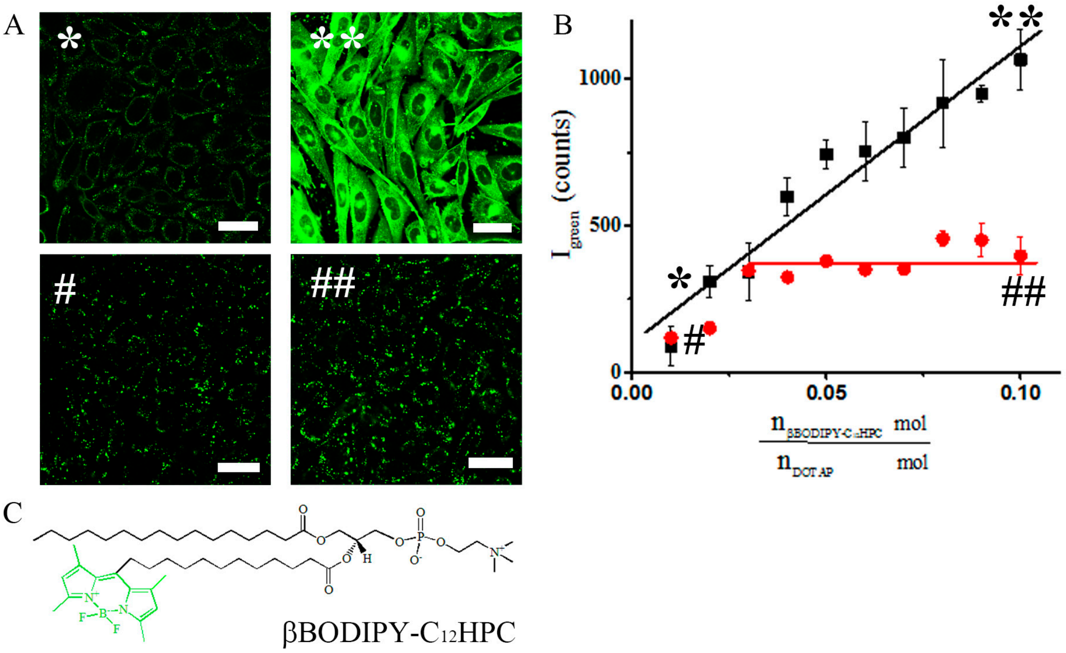

Figure 1. Importance of the aromatic component. (**A**) Fluorescence micrographs of CHO cells treated with liposomes containing βBodipy-C_{12}HPC as aromatic component in FLs at 0.01 (*) and 0.1 (**) mol/mol concentration as well as in ELs at the same concentrations (# and ##). Scale bars, 20 μm. (**B**) Dependence of fusion efficiency on dye concentration in FLs (black squares) and in ELs (red circles). Measurement points with standard deviations are shown. Lines represent linear fits. (**C**) Molecular structure of the chain labeled lipid βBodipy-C_{12}HPC. The green color of the aromatic molecular part represents the spectral emission of the fluorophore.

International Journal of
Molecular Sciences

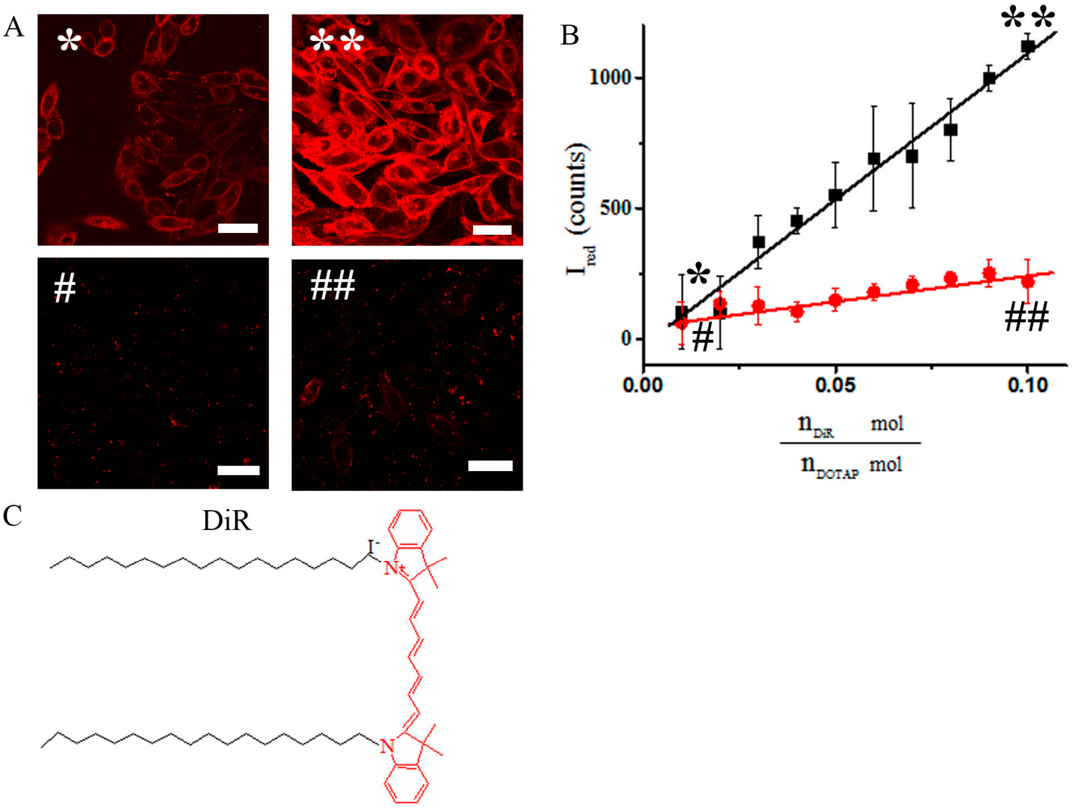

Figure 2. Importance of the aromatic component. (**A**) Fluorescence micrographs of CHO cells treated with liposomes containing DiR as aromatic component in FLs at 0.01 (∗) and 0.1 (∗∗) mol/mol concentration as well as in ELs at the same concentrations (# and ##). Scale bars, 20 µm. (**B**) Dependence of fusion efficiency on dye concentration in FLs (black squares) and in ELs (red circles). Measurement points with standard deviations are shown. Lines represent linear fits. (**C**) Molecular structure of DiR. The red color of the aromatic molecular part represents the spectral emission of the fluorophore.

International Journal of *Molecular Sciences*

MDPI

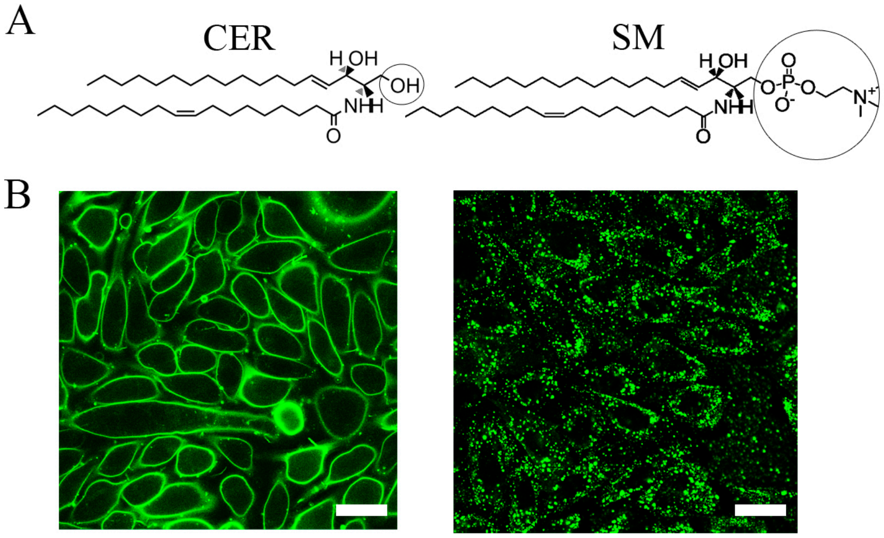

Figure 3. (**A**) Chemical structures of the neutral lipids N-oleoyl-D-erythro-sphingosine ceramide (CER) and N-oleoyl-D-erythro-sphingosylphosphorylcholine (SM). (**B**) Fluorescence micrographs of CHO cells treated with liposomes containing CER or SM as neutral lipid, DOTAP as cationic lipid, and BODIPY FL-DHPE as fluorescent component (1/1/0.1 mol/mol). Scale bars, 20 μm.

International Journal of Molecular Sciences

MDPI

Table 1. Composition of all liposomes used in our experiments.

Experiment	Neutral lipid	Cationic lipid	Aromatic component	Non-aromatic component	Molar ratio
1	DOPE	DODAP	BODIPY FL-DHPE	-	1/1/0.1
1	DOPE	DOTAP	BODIPY FL-DHPE	-	1/1/0.1
2	DOPE	DOTAP	BODIPY FL-DHPE	-	2/0/0.1
2	DOPE	DOTAP	BODIPY FL-DHPE	-	1/0.5/0.1
2	DOPE	DOTAP	BODIPY FL-DHPE	-	1/1/0.1
2	DOPE	DOTAP	BODIPY FL-DHPE	-	0/2/0.1
3	DOPE	DOTAP	BODIPY FL-DHPE	-	1/1/0.1
3	DOPE	DOTMA	BODIPY FL-DHPE	-	1/1/0.1
3	DOPE	DMTAP	BODIPY FL-DHPE	-	1/1/0.1
3	DOPE	DOEPC	BODIPY FL-DHPE	-	1/1/0.1
3	DOPE	DC-Cholesterol	BODIPY FL-DHPE	-	1/1/0.1
3	DOPE	MVL5	BODIPY FL-DHPE	-	1/1/0.1
4	DOPE	DOTAP	BODIPY FL-DHPE	-	1/1/0.01
4	DOPE	DOTAP	BODIPY FL-DHPE	-	1/1/0.02
4	DOPE	DOTAP	BODIPY FL-DHPE	-	1/1/0.03
4	DOPE	DOTAP	BODIPY FL-DHPE	-	1/1/0.04
4	DOPE	DOTAP	BODIPY FL-DHPE	-	1/1/0.05
4	DOPE	DOTAP	BODIPY FL-DHPE	-	1/1/0.06
4	DOPE	DOTAP	BODIPY FL-DHPE	-	1/1/0.07
4	DOPE	DOTAP	BODIPY FL-DHPE	-	1/1/0.08
4	DOPE	DOTAP	BODIPY FL-DHPE	-	1/1/0.09
4	DOPE	DOTAP	BODIPY FL-DHPE	-	1/1/0.1
5	DOPE	DOTAP	βBodipy-C12HPC	-	1/1/0.01
5	DOPE	DOTAP	βBodipy-C12HPC	-	1/1/0.02
5	DOPE	DOTAP	βBodipy-C12HPC	-	1/1/0.03
5	DOPE	DOTAP	βBodipy-C12HPC	-	1/1/0.04
5	DOPE	DOTAP	βBodipy-C12HPC	-	1/1/0.05
5	DOPE	DOTAP	βBodipy-C12HPC	-	1/1/0.06
5	DOPE	DOTAP	βBodipy-C12HPC	-	1/1/0.07
5	DOPE	DOTAP	βBodipy-C12HPC	-	1/1/0.08
5	DOPE	DOTAP	βBodipy-C12HPC	-	1/1/0.09
5	DOPE	DOTAP	βBodipy-C12HPC	-	1/1/0.1
6	DOPE	DOTAP	DiR	-	1/1/0.01
6	DOPE	DOTAP	DiR	-	1/1/0.02
6	DOPE	DOTAP	DiR	-	1/1/0.03
6	DOPE	DOTAP	DiR	-	1/1/0.04
6	DOPE	DOTAP	DiR	-	1/1/0.05
6	DOPE	DOTAP	DiR	-	1/1/0.06
6	DOPE	DOTAP	DiR	-	1/1/0.07
6	DOPE	DOTAP	DiR	-	1/1/0.08
6	DOPE	DOTAP	DiR	-	1/1/0.09
6	DOPE	DOTAP	DiR	-	1/1/0.1
7	DOPC	DOTAP	BODIPY FL-DHPE	-	1/1/0.01
7	DOPC	DOTAP	BODIPY FL-DHPE	-	1/1/0.02
7	DOPC	DOTAP	BODIPY FL-DHPE	-	1/1/0.03
7	DOPC	DOTAP	BODIPY FL-DHPE	-	1/1/0.04
7	DOPC	DOTAP	BODIPY FL-DHPE	-	1/1/0.05
7	DOPC	DOTAP	BODIPY FL-DHPE	-	1/1/0.06
7	DOPC	DOTAP	BODIPY FL-DHPE	-	1/1/0.07
7	DOPC	DOTAP	BODIPY FL-DHPE	-	1/1/0.08
7	DOPC	DOTAP	BODIPY FL-DHPE	-	1/1/0.09

7	DOPC	DOTAP	BODIPY FL-DHPE	-	1/1/0.1
8	DOPC	DOTAP	βBodipy-C_{12}HPC	-	1/1/0.01
8	DOPC	DOTAP	βBodipy-C_{12}HPC	-	1/1/0.02
8	DOPC	DOTAP	βBodipy-C_{12}HPC	-	1/1/0.03
8	DOPC	DOTAP	βBodipy-C_{12}HPC	-	1/1/0.04
8	DOPC	DOTAP	βBodipy-C_{12}HPC	-	1/1/0.05
8	DOPC	DOTAP	βBodipy-C_{12}HPC	-	1/1/0.06
8	DOPC	DOTAP	βBodipy-C_{12}HPC	-	1/1/0.07
8	DOPC	DOTAP	βBodipy-C_{12}HPC	-	1/1/0.08
8	DOPC	DOTAP	βBodipy-C_{12}HPC	-	1/1/0.09
8	DOPC	DOTAP	βBodipy-C_{12}HPC	-	1/1/0.1
9	DOPC	DOTAP	DiR	-	1/1/0.01
9	DOPC	DOTAP	DiR	-	1/1/0.02
9	DOPC	DOTAP	DiR	-	1/1/0.03
9	DOPC	DOTAP	DiR	-	1/1/0.04
9	DOPC	DOTAP	DiR	-	1/1/0.05
9	DOPC	DOTAP	DiR	-	1/1/0.06
9	DOPC	DOTAP	DiR	-	1/1/0.07
9	DOPC	DOTAP	DiR	-	1/1/0.08
9	DOPC	DOTAP	DiR	-	1/1/0.09
9	DOPC	DOTAP	DiR	-	1/1/0.1
10	DOPE	DOTAP	BODIPY FL-DHPE	DiR	1/1/0.02/0.1
10	DOPE	DOTAP	BODIPY FL-DHPE	Cholesterol	1/1/0.02/0.1
10	DOPE	DOTAP	BODIPY FL-DHPE	Biotinylcap-DOPE	1/1/0.02/0.1
10	DOPE	DOTAP	BODIPY FL-DHPE	PEG2000-DOPE	1/1/0.02/0.1
11	DMPE	DOTAP	BODIPY FL-DHPE	-	1/1/0.1
11	DPPE	DOTAP	BODIPY FL-DHPE	-	1/1/0.1
11	DPaPE	DOTAP	BODIPY FL-DHPE	-	1/1/0.1
11	DSPE	DOTAP	BODIPY FL-DHPE	-	1/1/0.1
11	DOPE	DOTAP	BODIPY FL-DHPE	-	1/1/0.1
11	LysoPE	DOTAP	BODIPY FL-DHPE	-	1/1/0.1
12	DMPC	DOTAP	BODIPY FL-DHPE	-	1/1/0.1
12	DPPC	DOTAP	BODIPY FL-DHPE	-	1/1/0.1
12	DPaPC	DOTAP	BODIPY FL-DHPE	-	1/1/0.1
12	DSPC	DOTAP	BODIPY FL-DHPE	-	1/1/0.1
12	DOPC	DOTAP	BODIPY FL-DHPE	-	1/1/0.1
12	DLiPC	DOTAP	BODIPY FL-DHPE	-	1/1/0.1
12	DEPC	DOTAP	BODIPY FL-DHPE	-	1/1/0.1
12	LysoPC	DOTAP	BODIPY FL-DHPE	-	1/1/0.1
12	SM	DOTAP	BODIPY FL-DHPE	-	1/1/0.1
12	CER	DOTAP	BODIPY FL-DHPE	-	1/1/0.1

International Journal of
Molecular Sciences

MDPI

Table 2. Characteristics of liposomes containing non-aromatic molecules as third components and DOPE/DOTAP/BODIPY FL-DHPE (0.1/1/1/0.02 mol/mol). The aromatic BODIPY FL-DHPE was used below its critical fusogenic concentration. Fusion efficiency of liposomes with CHO cells, as well as liposomal zeta potential (ς), and hydrodynamic size (d) are listed.

Lipids	Fusion eff. (s.d.) (%)	ς (s.d.) (mV)	d (s.d) (nm)
Biotinylcap-DOPE	0 (0)	59 (7)	116 (29)
PEG2000-DOPE	8 (2)	64 (5)	165 (31)
Cholesterol	0 (0)	65 (4)	124 (19)
DiR	90 (7)	64 (3)	165 (2)
